# Stable isotopes document the winter foraging ecology of king penguins and highlight connectivity between subantarctic and Antarctic ecosystems

**DOI:** 10.1002/ece3.3883

**Published:** 2018-02-08

**Authors:** Yves Cherel, Charline Parenteau, Paco Bustamante, Charles‐André Bost

**Affiliations:** ^1^ Centre d'Etudes Biologiques de Chizé (CEBC) UMR 7372 du CNRS‐Université de La Rochelle Villiers‐en‐Bois France; ^2^ Littoral, Environnement et Sociétés (LIENSs) UMR 7266 du CNRS‐Université de la Rochelle La Rochelle France

**Keywords:** diet, myctophid, prolactin, seabird, Southern Ocean

## Abstract

The poorly known winter foraging ecology of the king penguin, a major Southern Ocean consumer, was investigated at the subantarctic Crozet Islands where the largest global population breeds. Blood δ^13^C and δ^15^N values were used as proxies of the birds’ foraging habitat and diet, respectively, and circulating prolactin levels helped in determining the birds’ reproductive status. Plasma prolactin concentrations showed that king penguin adults of unknown breeding status (*n* = 52) that were present at the colony in winter were in fact breeders and failed breeders, but were not non ‐breeders. Circulating prolactin was neither related to δ^13^C nor δ^15^N values, thus suggesting that both breeders and failed breeders used the same foraging habitats and fed on the same prey. Plasma and blood cell isotopic values depicted four new relevant biological features on the feeding strategies of king penguins during the critical winter period: (1) 42% of the birds foraged in the distant Antarctic Zone, but 58% fed primarily in subantarctic waters (δ^13^C), (2) they preyed upon myctophids in both zones (δ^15^N), (3) individuals were consistent in their foraging strategies over the winter months (δ^13^C and δ^15^N), and (4) a higher proportion of females (77%–80%) than males (27%–31%) favored feeding in distant Antarctic waters (δ^13^C). This study highlights trophic connectivity between subantarctic and Antarctic ecosystems and hence the key role of energy export from Antarctic waters to sustain breeding populations of subantarctic predators, including during the Austral winter.

## 1 INTRODUCTION

Environmental variability influences avian population dynamics. In seabirds, climatic variations during winter affect fluctuations in population numbers, in many cases operating on variation in adult survival (Barbraud & Weimerskirch, 2003; Grosbois & Thompson, 2005; Sandvik, Erikstad, Barrett, & Yoccoz, 2005). A major challenge in identifying the underlying biological mechanisms is the lack of knowledge on foraging habits during winter, when most seabirds disperse far from their breeding grounds. The lack of winter habitat‐use and dietary information is particularly relevant for penguins, because the swimming and diving behavior of these flightless predators make them cryptic organisms when at sea. Consequently, little is known about their winter biology, although increasing use of bio‐logging has provided new insights into their diving patterns (Green, Boyd, Woakes, Warren, & Butler, 2005) and foraging grounds (Ballard et al., 2010; Bost, Thiébot, Pinaud, Cherel, & Trathan, 2009). This lack of information is of special concern, because (1) penguins number ~113 millions of individuals (van Franeker, Bathmann, & Mathot, 1997) and form 90% of seabird biomass in the Southern Ocean (Woehler, 1993), where they constitute a key group of marine consumers within the pelagic ecosystem (de Brooke, 2004; Woehler, 1995), and (2) populations of many species have declined substantially in the past two decades, with penguins being now the most threatened seabird taxon after albatrosses (Trathan et al., 2015).

**Figure 1:**
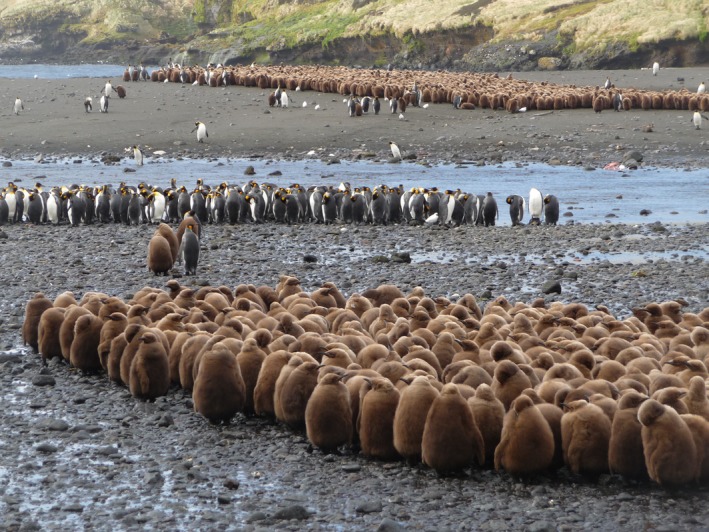
The king penguin colony of La Baie du Marin (Possession Island, Crozet Archipelago) in winter. Two crèches of large chicks are in the foreground and background, adults with unknown reproductive status rest near the river, and a few breeders with their corresponding single chicks stand in the periphery of the crèches. Small white birds are lesser sheathbills (*Chionis minor*)

The subantarctic king penguin is the sixth largest seabird consumer worldwide (de Brooke, 2004). The breeding cycle of the king penguin is unique amongst avian species since it spans >1 year, thus including the winter period. At that time, most adults desert the colonies and chicks fast (Figure 1), being fed at irregular intervals by the returning parents (Saraux, Friess, Le Maho, & Le Bohec, 2012). King penguins prey upon myctophids in spring, summer and autumn (Cherel, Fontaine, Richard, & Labat, 2010; Cherel, Verdon, & Ridoux, 1993; Raclot, Groscolas, & Cherel, 1998), but the diet of chicks indicates a shift to more diverse fish prey and to cephalopods in winter (Cherel, Ridoux, & Rodhouse, 1996; Moore, Robertson, & Wienecke, 1998). Winter is also the key period driving population dynamics of the species, because adult mortality occurs almost exclusively at that time (Le Bohec et al., 2007; Olsson & van der Jeugd, 2002).

The king penguin is one of the most thoroughly studied seabirds as a behavioral, ecological, and physiological model of a colonial and diving animal (Aubin & Jouventin, 1998; Bost et al., 2015; Handrich et al., 1997; Kooyman et al., 1992). However, despite the overwhelming biological importance of the winter season, most foraging studies related to this species focused on the summer months. At that time, breeding adults from the subantarctic Crozet Islands (its main breeding ground) forage mainly in the Subantarctic Zone (SAZ) to the Polar Front (PF) (Bost et al., 1997, 2015; Charrassin & Bost, 2001). In winter, no information is available on the at‐sea behavior of non breeders and failed breeders, but the few tracked breeders showed a different strategy. They perform very long trips to the south, crossing the PF and feeding for themselves in the Antarctic Zone (AZ) on still unknown prey (Bost, Charrassin, Clerquin, Ropert‐Coudert, & Le Maho, 2004; Charrassin & Bost, 2001; Pütz, 2002). However, trips were 30% longer for equipped than unequipped birds and desertion occurred in many cases (Bost et al., 2004); hence, more investigations using complementary methods are needed to record the foraging behavior of king penguins during the critical winter period.

The main goal of the present work was to document the food and feeding ecology of king penguins during the Austral winter using the stable isotope method (δ^13^C and δ^15^N) on a representative numbers (~50) of adult birds of unknown breeding status (hereafter UBS birds) that are found in the colony at that time (Figure 1). Reproductive status of UBS penguins (presumably a pool of breeders, failed breeders, and non breeders) was *a posteriori* investigated by measuring their plasma concentration of prolactin, a hormone involved in parental care in birds (Angelier, Wingfield, Tartu, & Chastel, 2016). The use of δ^13^C and δ^15^N values was validated on consumers from the southern Indian Ocean. Tissue δ^13^C values of predators reflect the decreasing δ^13^C gradient at the base of the food web from the tropics to Antarctic waters and thus indicate the predator latitudinal foraging habitat (Cherel & Hobson, 2007; Jaeger, Lecomte, Weimerskirch, Richard, & Cherel, 2010). Tissue δ^15^N values of consumers change according to their trophic position in the increasing order crustacean eaters < myctophid eaters < fish and squid eaters (Cherel et al., 2010). This method complements bio‐logging, with the main advantage that initial marking of individuals is not necessary and that every capture will provide foraging information *before* tissue sampling, and hence with no deleterious effect on the recorded behavior. We posed the following four predictions about the foraging habitat, diet, and sex‐specific strategies of UBS king penguins during the winter months.

1. *Prediction 1. King penguins forage in Antarctic waters in winter*. As previously tracked king penguins foraged within the AZ, most UBS birds would present low blood δ^13^C values that characterize feeding on Antarctic prey. Based on penguin blood isoscapes, δ^13^C values <−22.5 ‰, from −22.5 to −19.7 ‰, and >−19.7 ‰ were considered to correspond to the AZ, SAZ, and Subtropical (STZ) zones, respectively (Cherel & Hobson, 2007; Jaeger, Lecomte, et al., 2010). Hence, δ^13^C values < −22.5 ‰ record foraging within the AZ.
2. *Prediction 2. King penguins feed on Antarctic krill in winter*. In the southern Indian Ocean, most tracked breeders reached the northern sea ice edge and beyond within the Seasonal Ice Zone (SIZ, as far south as 63–65°S; Moore, Wienecke, & Robertson, 1999; Charrassin & Bost, 2001; Pütz, 2002), where it was hypothesized that king penguins shift from a fish‐ to a krill‐based diet (Bost et al., 2004; Le Bohec et al., 2008). While tracking give no dietary information, blood δ^15^N values differentiate penguins that feed on low versus higher (krill vs. pelagic fish) trophic level prey (Cherel, 2008; Cherel, Hobson, Guinet, & Vanpé, 2007).
3. *Prediction 3. Individual king penguins show consistency in their winter foraging strategies*, a common behavior in many seabirds that are faithful to their wintering grounds (Phillips, Lewis, Gonzalez‐Solis, & Daunt, 2017). Comparison of δ^13^C and δ^15^N values in tissues that record trophic information at different time scales depicts trophic variations at the individual level (Hobson & Bond, 2012; Martinez del Rio, Sabat, Anderson‐Sprecher, & Gonzalez, 2009). Positive linear relationships between isotopic values in plasma (short‐term integration, days to weeks) versus red blood cells (RBC, medium‐term integration, weeks to months; Hobson & Clark, 1993; Barquete, Strauss, & Ryan, 2013) would indicate consistency in the foraging habitat and diet of individual king penguins over the winter months. Conversely, contrasted (unrelated) plasma and RBC values would highlight time‐related changes in their winter strategy (Cherel, Connan, Jaeger, & Richard, 2014).
4. *Prediction 4. Females forage more in Antarctic waters than males*. The rationale is that females perform fewer and longer foraging trips than males in winter (Descamps, Gauthier‐Clerc, Gendner, & Le Maho, 2002; Le Vaillant, Ropert‐Coudert, Le Maho, & Le Bohec, 2016; Saraux et al., 2012). Hence, females have more time than males to reach and feed within the distant AZ. Unfortunately, the sex of all but one of the previously winter‐tracked king penguins was unknown (Bost et al., 2004; Charrassin & Bost, 2001; Jouventin, Capdeville, Cuénot‐Chaillet, & Boiteau, 1994; Moore et al., 1999; Pistorius et al., 2017; Pütz, 2002).

## 2 MATERIALS AND METHODS

The study took place at the king penguin colony of La Baie du Marin, Possession Island, Crozet Archipelago. The Crozet Islands (46–47°S) are located within the SAZ of the southern Indian Ocean. The Southern Ocean is here defined as the ocean south of the Subtropical Front (STF), and the AZ, SAZ, and STZ as the zones south of the Polar Front (PF), between the PF and STF, and north of the STF, respectively.

Fieldwork was carried out during two Austral winters (2002 and 2008). At the very end of winter 2002 (early September), randomly chosen breeding adults (*n* = 10) and their corresponding chicks were blood sampled, and the adults were stomach‐flushed one time to collect a representative subsample (100–400 g) of the food provisioned to their single offspring. In winter 2008 (July‐August), three groups of birds were blood sampled: chicks (*n* = 10), breeders (*n* = 9), and adults of unknown status (UBS birds, *n* = 52). Blood was collected into a heparinized syringe by venepuncture of a flipper vein. In 2008 (not 2002), whole blood was centrifuged to separate plasma from RBC. Blood and food samples were kept frozen at −20°C until analysis in France.

Food samples were thawed overnight over a sieve to remove the liquid fraction. The solid fraction was then placed in a large flat‐bottomed tray, and fresh remains were divided into broad prey classes (fish and cephalopods), which were weighed to estimate their proportions by mass in the diet. Total numbers of each prey item were counted in each individual food sample. Prey was identified using published keys and descriptions and by comparison with material (squid beaks and fish bones and otoliths) held in our own reference collection (Cherel et al., 1996; Xavier & Cherel, 2009).

Tissue δ^13^C and δ^15^N values were determined on food samples, and, depending on study groups, on whole blood, RBC, and plasma (Table 1). Food and blood samples were freeze‐dried and powdered. King penguins feed on fatty fishes (Raclot et al., 1998) and plasma, unlike whole blood and RBC, contains a high and variable lipid content that affect its δ^13^C values (Cherel, Hobson, Bailleul, & Groscolas, 2005; Cherel, Hobson, & Hassani, 2005; Cherel, Hobson, & Weimerskirch, 2005). C:N mass ratios indicated that cyclohexane efficiently extracted lipids from fish muscle (Cherel et al., 2010), but not from plasma (Cherel, Connan, et al., 2014), which thus required a stronger delipidation using chloroform/methanol (Cherel, Hobson, & Weimerskirch, 2005). Tissue sub samples were weighed with a microbalance, packed in tin containers, and nitrogen and carbon isotope ratios were subsequently determined by a continuous flow mass spectrometer (Thermo Scientific Delta V Advantage) coupled to an elemental analyzer (Thermo Scientific Flash EA 1112). Results are presented in the usual δ notation relative to Vienna PeeDee Belemnite and atmospheric N_2_ for δ^13^C and δ^15^N, respectively. Replicate measurements of internal laboratory standards (acetanilide and peptone) indicate measurement errors <0.15 ‰ for both δ^13^C and δ^15^N values.

**Table 1:** Food and blood δ^13^C and δ^15^N values of king penguins during the Austral winter. Lipids were removed from food samples and plasma, but not from RBC and whole blood (see text)

Groups	*n*	Tissue	δ^13^C (‰)	δ^15^N (‰)	C:N mass ratio
Winter 2002					
Food samples	10	Digested fish	−22.4 ± 0.3 (−22.7 to −21.8)	7.3 ± 0.3 (6.9–7.7)	3.68 ± 0.10
Chicks	10	Whole blood	−21.2 ± 0.2 (−21.6 to −20.9)	11.1 ± 0.3 (10.6–11.5)	3.47 ± 0.05
Breeders	10	Whole blood	−21.9 ± 0.4 (−22.4 to −21.2)	9.9 ± 0.4 (9.4–10.5)	3.45 ± 0.04
Winter 2008					
Chicks	10	Whole blood	−21.9 ± 0.5 (−22.4 to −20.9)	10.7 ± 0.3 (10.4–11.3)	3.38 ± 0.07
Breeders	9	Blood cells	−21.4 ± 0.6 (−22.1 to −20.5)	10.0 ± 0.2 (9.8–10.4)	3.26 ± 0.03
	8	Plasma	−21.5 ± 0.7 (−22.4 to −20.8)	10.7 ± 0.3 (10.2–11.2)	3.45 ± 0.05
UBS adults	52	Blood cells	−22.3 ± 0.6 (−23.5 to −21.2)	10.3 ± 0.4 (9.1–11.0)	3.26 ± 0.04
	48	Plasma	−22.2 ± 0.8 (−23.9 to −20.7)	11.1 ± 0.4 (9.9–11.8)	3.43 ± 0.05

UBS, unknown breeding status (see text).

Values are means ± *SD* with ranges in parentheses.

Sexing UBS king penguins is challenging because (1) unlike breeders, they do not perform their sex‐specific call to communicate with their single chicks or mates (Derenne, Jouventin, & Mougin, 1979), and (2) the species is only slightly dimorphic, with males being larger than, but overlapping in size with females (Barrat, 1976). Hence, UBS birds and breeders from 2008 were genetically sexed using a molecular method (Fridolfsson & Ellegren, 1999) on either whole blood or RBC. Prolactin concentration was measured using a heterologous radioimmunoassay on plasma (Cherel, Mauget, Lacroix, & Gilles, 1994) to determine the breeding status of UBS adults sampled in winter 2008. In king penguins, circulating prolactin is high during incubation and the long chick‐rearing period that includes winter; it remains at moderate levels in failed breeders but is low during molt, pairing, and courtship (Cherel et al., 1994; Garcia, Jouventin, & Mauget, 1996; Jouventin & Mauget, 1996).

Data were statistically analyzed using SYSTAT 13. Values are means ± *SD*. δ^13^C, and δ^15^N values of whole blood were considered to be comparable to those of RBC, because RBC contains more organic matter than plasma and consequently whole blood has δ^13^C and δ^15^N values very close to those of RBC (Cherel, Hobson, Bailleul, et al., 2005). To help interpret blood δ^13^C and δ^15^N values of king penguins, they were compared to blood values of the two truly Antarctic penguins, the emperor and Adélie penguins, which forage at high latitudes and feed mainly on pelagic fish and krill, respectively (Cherel, 2008).

## 3 RESULTS

In late winter 2002, the food of king penguin chicks was dominated by fish (98.7% and 97.8% by mass and number, respectively), with cephalopods accounting for the remaining 1.3%–2.2%. Fishes were primarily myctophids (94.2% by number). Two species, *Krefftichthys anderssoni* and *Protomyctophum tenisoni*, were present in all the food samples (*n* = 10) and almost equally formed the main part of the diet (49.0% and 44.3% by number, respectively). Two other prey were found in most samples (*n* = 8), namely the paralepidid fish *Arctozenus risso* (3.1%) and the onychoteuthid squid *Kondakovia longimana* (1.5%).

**Figure 2:**
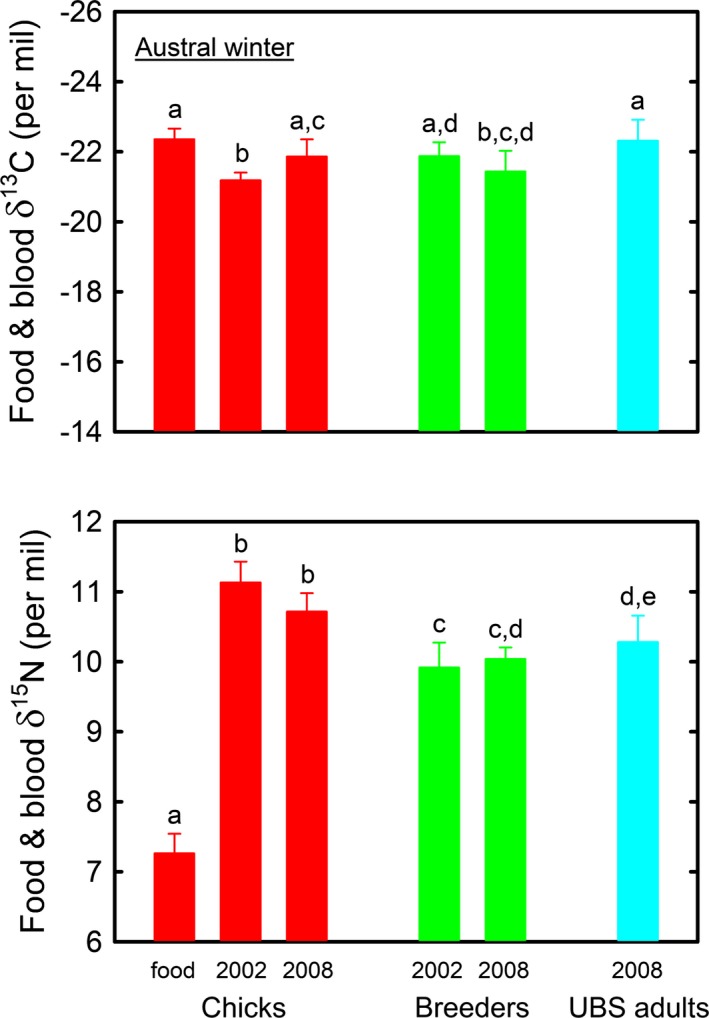
Food and blood δ^13^C (upper panel) and δ^15^N (lower panel) values of king penguins during the Austral winter. Values not sharing the same superscripted letter are significantly different at *p *<* *.05 (Tukey's Honestly‐Significant‐Difference tests). Values are means ± *SD*. UBS, unknown breeding status (see text)

Food samples and groups of king penguins (whole blood or RBC) segregated by both their δ^13^C and δ^15^N values (ANOVA, *F*_5,95_ = 11.97 and 174.27, respectively, both *p *<* *.0001). δ^15^N value was lower in food samples than in blood (Tukey's Honestly‐Significant‐Difference tests, all *p *<* *.0001) (Figure 2). Blood δ^15^N values varied slightly among penguin groups, with chicks being significantly ^15^N‐enriched when compared to breeders and UBS adults (all *p* ≤ .004). Overall the range of mean δ^13^C values was low. In contrast to breeders and chicks, some UBS birds presented very ^13^C‐depleted values (Table 1). Accordingly, mean plasma δ^13^C value was lower in UBS adults than in breeders sampled in winter 2008 (two‐sample *t*‐test, *t *=* *2.45, *p *=* *.018).

Most breeders sampled in winter 2008 were males (8 of 9). Sex ratio also differed significantly from parity amongst the 52 sampled UBS adults, which included more males than females (71% vs. 29%, respectively; single proportion test, *Z *=* *3.05, *p *=* *.002). Plasma prolactin concentration (range: 25–115 ng/ml) was significantly higher in UBS females than males (Table 2), and it was higher in breeding males than in UBS males (*n* = 7 and 35, 54 ± 13 and 42 ± 10 ng/ml, Mann–Whitney *U*‐test, *U *=* *187.0, *p *=* *.029). Circulating prolactin in UBS females and males showed a continuous range of values that were not linearly correlated with either plasma δ^13^C or δ^15^N values (least squares method and associated ANOVA, females: *F*_1,11_ = 0.08 and 0.50, *p *=* *.785 and 0.495; males: *F*_1,33_ = 0.16 and 4.15, *p *=* *.693 and 0.050, respectively).

**Table 2:** Blood δ^13^C and δ^15^N values and circulating prolactin of UBS king penguins during the Austral winter 2008. See text for isotopic delineation of water masses, and thus of birds foraging either in the Antarctic or Subantarctic zones

	Females	Males	Two sample *t*‐tests	
			*t*	*p*
Red blood cells				
All UBS adults				
*n*	15	37		
δ ^13^C (‰)	−**22.8 ± 0.5**	−**22.1 ± 0.5**	**3.97**	**<.0001**
δ ^15^N (‰)	10.3 ± 0.4	10.3 ± 0.4	0.09	.928
C:N mass ratio	3.26 ± 0.03	3.26 ± 0.05	0.24	.810
Antarctic zone				
*n*	12	10		
δ ^13^C (‰)	−22.9 ± 0.3	−22.8 ± 0.3	0.84	.409
δ ^15^N (‰)	10.3 ± 0.4	10.4 ± 0.2	0.62	.543
Subantarctic zone				
*n*	3	27		
δ ^13^C (‰)	−22.1 ± 0.5	−21.9 ± 0.4	na	na
δ ^15^N (‰)	10.2 ± 0.3	10.2 ± 0.4	na	na
Plasma				
All UBS adults				
*n*	13	35		
δ ^13^C (‰)	−**22.9 ± 0.6**	−**22.0 ± 0.8**	**4.14**	**<.0001**
δ ^15^N (‰)	11.1 ± 0.4	11.1 ± 0.4	0.01	.996
C:N mass ratio	3.43 ± 0.04	3.43 ± 0.06	0.15	.880
Prolactin (ng/ml)	**77 ± 26**	**42 ± 10**	**6.84**	**<.0001**
Antarctic Zone				
*n*	10	11		
δ ^13^C (‰)	−23.2 ± 0.3	−22.9 ± 0.4	2.06	.053
δ ^15^N (‰)	11.3 ± 0.2	11.3 ± 0.2	0.41	.683
Prolactin (ng/ml)	**77 ± 28**	**40 ± 10**	**4.15**	**.001**
Subantarctic Zone				
*n*	3	24		
δ ^13^C (‰)	−22.0 ± 0.1	−21.6 ± 0.4	na	na
δ ^15^N (‰)	10.5 ± 0.3	11.1 ± 0.5	na	na
Prolactin (ng/ml)	77 ± 23	43 ± 11	na	na

Statistical differences are marked in bold. Values are means ± *SD*.

UBS, unknown breeding status (see text).

**Figure 3:**
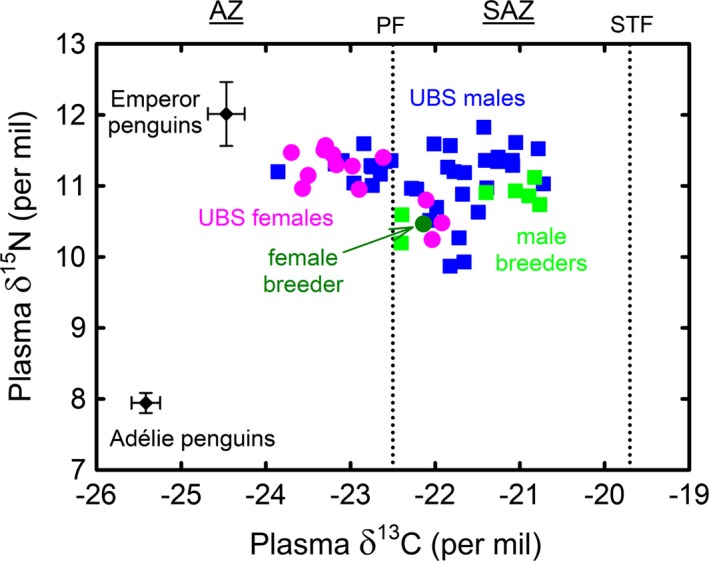
Delipidated plasma δ^15^N versus δ^13^C values in UBS adults and breeders of king penguins sampled in the Austral winter 2008. Whole blood isotopic values of two high‐Antarctic seabirds that feed primarily on fish (breeding emperor penguins in spring) and crustaceans (breeding Adélie penguins in spring) were included in the figure to help interpreting king penguin δ^13^C and δ^15^N values (Cherel, 2008). Following Cherel and Hobson (2007), dashed lines correspond to the δ^13^C estimation of the Polar Front (PF) and of the Subtropical Front (STF), which delimit the Antarctic (AZ), Subantarctic (SAZ), and Subtropical Zones. UBS, unknown breeding status (see text)

No sex‐related differences were found in either RBC or plasma δ^15^N values of UBS adults, but RBC and plasma δ^13^C values were significantly lower in females than in males (Table 2). Both RBC and plasma δ^13^C values indicated that proportionally more UBS females than males foraged within the AZ (RBC: 12 of 15 [80%] vs. 10 of 37 [27%], plasma: 10 of 13 [77%] vs. 11 of 35 [31%], equality of proportion tests, *Z *=* *3.50 and 2.82, *p *<* *.0001 and *p* = .005, respectively). Conversely, their blood δ^13^C values showed that more males than females foraged in the SAZ (Figure 3). When foraging within the same oceanographic zone, there were no significant differences in RBC and plasma δ^13^C and δ^15^N values between UBS males and females (Table 2).

**Figure 4:**
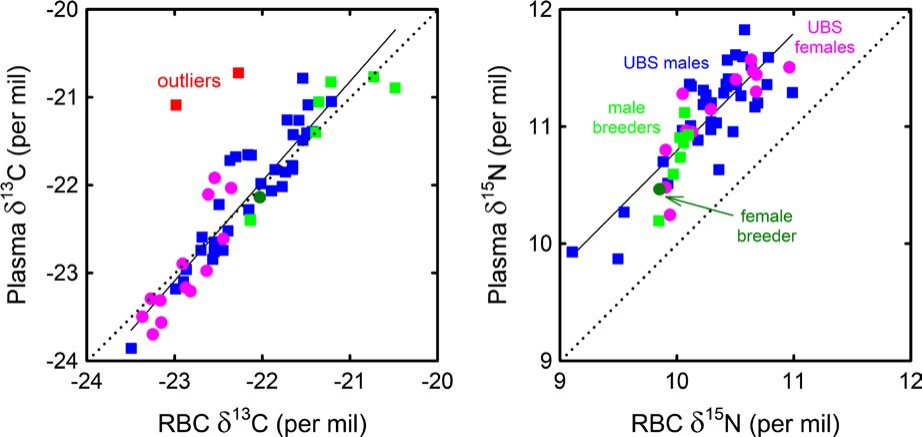
Delipidated plasma δ^13^C versus RBC δ^13^C values (left panel) and delipidated plasma δ^15^N versus RBC δ^15^N values (right panel) in UBS adults and breeders of king penguins sampled in the Austral winter 2008 (for outliers, see text). Regression lines (least squares method and associated ANOVA): δ^13^C (without outliers), *y* = 1.13*x* + 3.00, *F*_1,52_ = 345.83, *p *<* *.0001; δ^15^N, *y* = 1.01*x* + 0.74, *F*_1,54_ = 121.31, *p *<* *.0001. UBS, unknown breeding status (see text)

Plasma and RBC δ^13^C values were positively and linearly correlated in UBS adults and breeders sampled in winter 2008 (except two outliers; see discussion). Plasma and RBC δ^15^N values were also positively and linearly related among individuals of the two groups (Figure 4). Paired *t*‐tests indicated that δ^13^C values in RBC and plasma were not significantly different (*n* = 54 [without the two outliers], *t *=* *0.43, *p *=* *.667), but δ^15^N values were on average 0.80 ± 0.24 ‰ higher in plasma than in RBC (*n* = 56, *t *=* *24.51, *p *<* *.0001).

## 4 DISCUSSION

The present work exemplifies the usefulness of stable isotopes to complement the direct methods of bio‐logging and dietary analysis to reveal previously unknown foraging strategies during cryptic stages in the life cycle of animals (Cherel, Kernaléguen, Richard, & Guinet, 2009). Blood δ^13^C and δ^15^N values depict four relevant biological features on the foraging ecology of king penguins during the critical winter period: (1) overall, birds forage both in Antarctic (AZ) and subantarctic (SAZ) waters, (2) they primarily prey upon mesopelagic fish in both zones, (3) individuals are consistent in their foraging strategies over the winter months and (4) females have a greater tendency than males to feed in distant Antarctic waters.

Despite their long breeding cycle (>1 year), king penguins attempt to breed every year but none breed successfully in two consecutive seasons. Early breeders that are successful on year 1 become late breeders that fail during incubation or during the early chick‐rearing period on year 2, being thus again early breeders on year 3 (van Heezik, Seddon, Cooper, & Plös, 1994; Le Bohec et al., 2007; Olsson, 1996). Accordingly, both breeders and failed breeders are commonly found in the colony in winter (Descamps et al., 2002). Circulating prolactin in our study animals confirmed that pattern, with no UBS king penguins sampled in 2008 having low hormone levels that characterize non breeders (Garcia et al., 1996). This suggests that the few non breeders disperse widely and remain at sea without coming ashore, as most oceanic penguins (Bost et al., 2009; Thiébot, Cherel, Trathan, & Bost, 2012). Plasma prolactin concentration was elevated in all sampled UBS king penguins, and it was higher in females than in males, which is in agreement with previous investigations (Cherel et al., 1994; Garcia et al., 1996). No bimodality occurred in hormone levels, thus precluding a clear‐cut differentiation between breeders and failed breeders. Instead, the continuum of hormone values indicates that sampled UBS birds included unknown proportions of breeders and failed breeders. Within that context, a relevant finding is that circulating prolactin was neither related to blood δ^13^C nor δ^15^N values, thus suggesting that both breeders and failed breeders used the same foraging habitats and fed on the same prey during the winter months.

### 4.1 Prediction 1. King penguins forage in Antarctic waters in winter

King penguin chicks and breeders that were sampled in the winters of 2002 and 2008 showed limited variations in their isotopic values. Blood δ^13^C values indicated foraging within the SAZ and at the PF, which is in agreement with the many birds sampled at the Crozet Islands during the spring and summer months (Cherel, Hobson, Bailleul, et al., 2005; Cherel et al., 2007; Cherel, Y., unpublished data). Mean δ^13^C values of UBS adults sampled in winter 2008 are close to those of chicks and breeders, but they showed a larger range of individual values that illustrates three notable features about their feeding grounds:

1. Based on the known latitudinal δ^13^C gradient, low blood δ^13^C values indicated Antarctic foraging grounds for the species. Forty‐two percent of UBS adults foraged within the AZ, with six birds having RBC δ^13^C values <−23 ‰. However, an isotopic comparison with the two true Antarctic penguins, the emperor and Adélie penguins, shows that king penguins did not reach high‐Antarctic waters (Figure 3). Overall, the isotopic data are in close agreement with the few winter‐tracked breeders that forage within the SIZ, but not in dense pack ice further south (Bost et al., 2004; Charrassin & Bost, 2001; Pistorius et al., 2017; Pütz, 2002).
2. No UBS adults foraged in the subtropics, which confirms the many satellite‐tracking and isotopic investigations over sites and years that never showed any king penguin crossing the STF in the southern Indian Ocean (Bost et al., 1997; Charrassin & Bost, 2001; Cherel et al., 2007; Pistorius et al., 2017; Pütz, 2002).
3. Instead, both RBC and plasma δ^13^C showed a continuum of values indicating that winter foraging grounds of king penguins ranged from subantarctic to Antarctic waters. Importantly, blood δ^13^C values of a majority of UBS adults (58%) were similar to those found during summer, thus indicating that birds foraged primarily within the SAZ and at the PF in winter. In contrast, all but one winter satellite‐tracked breeders crossed the PF and foraged within the AZ (Bost et al., 2004; Moore et al., 1999; Pistorius et al., 2017; Pütz, 2002). This mismatch, together with dietary indication of feeding very close to the colony to feed the chicks (Cherel et al., 1996), highlights the need of further investigations using a combination of isotopic and bio‐logging methods on the same individuals to better detail the various foraging strategies of king penguins during winter.

### 4.2 Prediction 2. King penguins feed on Antarctic krill in winter

When foraging within the SAZ and at the PF in winter, king penguin breeders fed primarily on myctophids for themselves, as indicated by their blood δ^15^N values that were identical to the many previous measurements performed on the species at the Crozet Islands (Cherel, Hobson, Bailleul, et al., 2005; Cherel et al., 2007) and elsewhere (Cherel, Pütz, & Hobson, 2002; Cherel et al., 2010). A myctophid‐based diet was verified by the stepwise lower δ^15^N value of food delivered to chicks in early September 2002 (Figure 2). At that time, food was mainly composed of two myctophid species (*K. anderssoni* and *P. tenisoni*) that form the bulk of the chick diet in spring (Cherel et al., 1993) and temporally correspond to an increase in parent feeding visits from early September onward (Saraux et al., 2012). The slightly higher δ^15^N values of chicks than breeders result from two cumulative isotopic explanations: (1) Chicks fast most of the time in winter, thus using endogenous protein reserves that induces a progressive ^15^N enrichment of their tissues (Barrat, 1976; Cherel, Stahl, & Le Maho, 1987; Cherel, Hobson, Bailleul, et al., 2005), and (2) winter food of chicks includes squids that have higher δ^15^N values than fish (Cherel, Ducatez, Fontaine, Richard, & Guinet, 2008; Cherel et al., 1996).

Blood δ^15^N values of UBS adults indicate they also fed on fish whatever their δ^13^C values and hence their foraging zones (Figure 3). Feeding on myctophids in both the SAZ and AZ did not verify the hypothesis that king penguins shift to Antarctic krill when foraging in Antarctic waters (Bost et al., 2004; Le Bohec et al., 2008). This new finding is supported by several isotopic arguments.

1. Penguins feeding on low trophic level prey, that is, swarming crustaceans, have low blood δ^15^N values that do not fit with the higher king penguin values. Those species include pygoscelid penguins feeding on Antarctic krill in the AZ (Juares, Santos, Mennucci, Coria, & Mariano‐Jelicich, 2016; Polito et al., 2016) and crested penguins preying upon euphausiids and hyperiids within the SAZ, including at the Crozet Islands (Cherel et al., 2007).
2. Seabirds feeding on high trophic level prey (large fish, squids, and carrion) have higher δ^15^N values than king penguins, as illustrated by large petrels and albatrosses (Blévin et al., 2013).
3. Instead, blood δ^15^N values of UBS king penguins foraging within the AZ are identical to those of birds foraging in the SAZ (Cherel et al., 2007; this study) and close to the values of other myctophid eaters, including Antarctic and subantarctic fur seals, elephant seals, and blue petrels (Cherel et al., 2007, 2008; Cherel, Connan, et al., 2014). Hence, the king penguin is a meso‐predator that specializes on myctophids all year long. Identifying fish species targeted by king penguin in the AZ remains to be investigated, but all penguin δ^15^N values notably were >9.9 and >11.0 ‰ in RBC and plasma, respectively (Figure 3). Taking into account the king penguin blood‐diet discrimination factor (the differences in isotopic composition between blood and diet, here 2.1 ‰; Cherel, Hobson, & Hassani, 2005), these relatively high δ^15^N values suggest king penguins fed less on the lower trophic level *Krefftichthys anderssoni* (6.6–7.6 ‰) and *P. tenisoni* (6.9–8.1 ‰), and more on *Electrona antarctica* (8.3–8.9 ‰), the most abundant myctophid in Antarctic waters (Hulley, 1990; Cherel et al., 2010, unpublished data).

### 4.3 Prediction 3. Individual king penguins show consistency in their winter foraging strategies

Another relevant finding is that king penguins (both UBS adults and breeders) showed individual consistency in their winter foraging strategies, as indicated by the highly significant linear relationships between RBC and plasma isotopic values (Xavier et al., 2017). Individual birds foraged within the same isotopic habitat (δ^13^C) where they fed on the same isotopic diet (δ^15^N) during the months preceding blood sampling (Figure 4). This means that individuals foraging in Antarctic waters did not feed significantly within the SAZ but, instead fasted and relied on their energy reserves built up in the AZ to cover the energetic cost of travelling to the colony and remaining ashore, a “capital” strategy similar to that of some male Antarctic fur seals (Cherel et al., 2009). Fidelity to feeding habitats and prey presumably increases foraging success in winter, being thus beneficial during a period marked by a decrease in the availability of marine resources. Two birds showed a different strategy, however (outliers in Figure 4). Their more positive δ^13^C values in plasma than in RBC indicate that they shifted to more northern feeding grounds over the last weeks before blood sampling. The two king penguins exemplify the foraging plasticity of Southern Ocean seabirds facing various environmental conditions at different spatio temporal scales (Cherel, Connan, et al., 2014; Tremblay & Cherel, 2003).

Plasma was enriched in ^15^N (but not in ^13^C) when compared to RBC (Figure 4). This plasma‐RBC difference is in agreement with the values calculated in captive birds feeding on isotopically controlled diets (Federer, Hollmen, Esler, Wooller, & Wang, 2010; Hahn, Hoye, Korthals, & Klaassen, 2012; Kurle et al., 2013). The δ^15^N differences arise from tissue‐specific isotopic discrimination factors due primarily to tissue‐specific protein, and hence amino acid, composition (Wolf, Carleton, & Martinez del Rio, 2009). Such tissue‐specific discrimination factors preclude comparing raw isotopic values of different tissues without correcting them first (Cherel, Jaquemet, Maglio, & Jaeger, 2014).

### 4.4 Prediction 4. More females than males forage in Antarctic waters

A last new finding is that, as expected, females rather than males foraged within the AZ in winter. Females feeding further away than males are likely the explanation of chicks being visited two times less by females than by males in winter (Descamps et al., 2002). This means that theoretically ~1/3 of breeders present at the colony are female birds, a biased sex ratio that fit well with the proportion of females observed among the sampled UBS adults in winter 2008 (29%). Why females favor distant foraging grounds is difficult to interpret. In seabirds, sex‐related segregation in food and feeding ecology is linked to sexual size dimorphism with the underlying mechanisms being related to sexual specialization in diet or habitat, or size‐mediated competitive exclusion (Phillips, McGill, Dawson, & Bearhop, 2011). Whatever the driving factor is for king penguin, the finding potentially has important demographic consequences. During normal years, survival rate is high and identical for both sexes (Le Bohec et al., 2007, 2008; Olsson & van der Jeugd, 2002), but females survive less well than males following a catastrophic year marked by food shortage (Olsson & van der Jeugd, 2002). The sex‐related difference in survival can be explained by the sex‐related difference in winter foraging grounds because (1) king penguin mortality is negatively affected by warm events within the AZ (Le Bohec et al., 2008), and (2) females forage more within the AZ than males (present study), thus resulting in a skewed sex ratio favoring males in the whole population (Olsson & van der Jeugd, 2002).

### 4.5 Trophic connectivity between subantarctic and Antarctic ecosystems

King penguins largely foraged in Antarctic waters where they fed on mesopelagic fish. Breeding north the PF and foraging south of it appears a common life history trait of many warm‐blooded vertebrates. Dietary investigation, satellite‐tracking and stable isotopes concur to indicate that many subantarctic marine mammals and seabirds foraged in the AZ during parts of their life cycle (Table 3). They belong to a large diversity of taxa (pinnipeds, penguins, albatrosses, and petrels), which show a large range in size (from diving petrels to elephant seals), in flying ability (from flightless to dynamic soaring), and in feeding methods (pursuit diving and surface seizing being the commonest). Ecologically, they include major consumers of the Southern Ocean both in terms of numbers and in terms of biomass, thus emphasizing the key role of the nutritional flux from Antarctic waters to sustain breeding populations of subantarctic predators. Hence, the PF does not act as a biogeographic barrier for pinnipeds and seabirds, and there is a strong trophic connectivity between the AZ oceanic and SAZ island ecosystems.

**Table 3:** Review of dietary, tracking, and stable isotopes evidences that pinnipeds and seabirds breeding in subantarctic islands (southern Indian Ocean) forage in Antarctic waters. Dietary bio‐indicators are two endemic Antarctic prey species, the Antarctic krill *Euphausia superba* and oceanic squid *Psychroteuthis glacialis*

Species	Breeding localities	Life stages	Diet	Tracking	Stable isotopes	References
Southern elephant seal	Marion	Post‐breeding, post‐molt		+		(McIntyre, Bornemann, Plötz, Tosh, & Bester, 2011; McIntyre, Tosh, Plötz, Bornemann, & Bester, 2010; McIntyre et al., 2011)
(*Mirounga leonina*)	Kerguelen	Post‐breeding, post‐molt		+	+	(Authier, Dragon, Cherel, & Guinet, 2012; Bailleul, Charrassin, Ezraty, et al., 2007; Bailleul, Charrassin, Monestiez, et al., 2007; Chaigne, Authier, Richard, Cherel, & Guinet, 2013; Labrousse et al., 2015)
Antarctic fur seal	Marion	Winter		+	+	(Arthur et al., 2016)
(*Arctocephalus gazella*)	Crozet	Breeding, all year long			+	(Cherel et al., 2007, 2009; Kernaléguen et al., 2012; 2016)
King penguin	Marion	Chick‐rearing (winter)		+		(Pistorius et al., 2017)
(*Aptenodytes patagonicus*)	Crozet	Chick‐rearing (winter)		+	+	(Charrassin & Bost, 2001; Pütz, 2002; Bost et al., 2004; present study)
Macaroni penguin	Marion	Pre‐molt		+	+	(Whitehead, Connan, Ropert‐Coudert, & Ryan, 2017; Whitehead, Kato, Ropert‐Coudert, & Ryan, 2016)
(*Eudyptes chrysolophus*)	Crozet	Pre‐molt, winter migration		+	+	(Thiébot, Cherel, Trathan, & Bost, 2011; Thiébot et al., 2014)
	Kerguelen	pre‐molt, winter migration		+		(Thiébot et al., 2011, 2014)
Rockhopper penguin	Marion	Pre‐molt		+	+	(Whitehead et al., 2016, 2017)
(*Eudyptes chrysocome filholi*)						
Wandering albatross	Crozet	Incubation		+	+	(Jaeger, Lecomte, et al., 2010; Lecomte et al., 2010; Weimerskirch, Salamolard, Sarrazin, & Jouventin, 1993; Weimerskirch et al., 2014)
(*Diomedea exulans*)	Kerguelen	Incubation		+		(Pinaud & Weimerskirch, 2007)
Black‐browed albatross	Kerguelen	Incubation		+		(Pinaud & Weimerskirch, 2007)
(*Thalassarche melanophris*)						
Grey‐headed albatross	Marion	Chick‐rearing, molt	+	+	+	(Connan, McQuaid, Bonnevie, Smale, & Cherel, 2014; Jaeger et al., 2013; Nel et al., 2001; Richoux, Jaquemet, Bonnevie, Cherel, & McQuaid, 2010)
(*Thalassarche chrysostoma*)	Kerguelen	Chick‐rearing	+			(Cherel, Weimerskirch, & Trouvé, 2002)
Light‐mantled sooty albatross	Marion	Chick‐rearing, molt	+		+	(Berruti & Harcus, 1978; Connan et al., 2014; Cooper & Klages, 1995; Jaeger et al., 2013)
(*Phoebetria palpebrata*)	Crozet	Chick‐rearing, molt	+	+	+	(Jaeger, Connan, Richard, & Cherel, 2010; Pinaud & Weimerskirch, 2007; Ridoux, 1994)
	Kerguelen	Molt			+	(Jaeger et al., 2013)
Sooty albatross	Marion	Chick‐rearing	+			(Berruti & Harcus, 1978; Cooper & Klages, 1995)
(*Phoebetria fusca*)	Crozet	Chick‐rearing	+		+	(Pinaud & Weimerskirch, 2007; Ridoux, 1994)
Southern giant petrel	Crozet	Incubation, chick‐rearing		+		(Thiers et al., 2014)
(*Macronectes giganteus*)						
White‐chinned petrel	Crozet	Incubation, chick‐rearing	+	+	+	(Catard, Weimerskirch, & Cherel, 2000; Connan, Cherel, & Mayzaud, 2007; Jaeger, Connan, et al., 2010; Ridoux, 1994)
(*Procellaria aequinoctialis*)	Kerguelen	Incubation, chick‐rearing	+	+	+	(Delord et al., 2010; Jaeger et al., 2013; Péron et al., 2010)
White‐headed petrel	Kerguelen	Chick‐rearing			+	(Blévin et al., 2013)
(*Pterodroma lessoni*)						
Kerguelen petrel	Crozet	Chick‐rearing	+			(Ridoux, 1994)
(*Aphrodroma brevirostris*)	Kerguelen	Chick‐rearing, molt			+	(Blévin et al., 2013; unpublished data)
Blue petrel	Crozet	Chick‐rearing	+			(Ridoux, 1994)
(*Halobaena coerulea*)	Kerguelen	All year long	+	+	+	(Cherel, Bocher, Trouvé, et al., 2002 ; Cherel, Connan, et al., 2014; Cherel et al., 2016; Connan, Mayzaud, Trouvé, Barbraud, & Cherel, 2008; Quillfeldt, Cherel, Delord, & Weimerskirch, 2015)
Antarctic prion	Kerguelen	Chick‐rearing, molt	+		+	(Cherel, Bocher, de Broyer, et al., 2002 ; Cherel et al., 2016; Weimerskirch, Fradet, & Cherel, 1999)
(*Pachyptila desolata*)						
Thin‐billed prion	Kerguelen	All year long	+	+	+	(Cherel, Bocher, de Broyer, et al., 2002 ; Cherel, Connan, et al., 2014; Cherel et al., 2016; Quillfeldt et al., 2015)
(*Pachyptila belcheri*)						
Common diving petrel	Kerguelen	Spring, molt			+	(Bocher, Cherel, & Hobson, 2000; Cherel, Phillips, Hobson, & McGill, 2006; Cherel, Connan, et al., 2014)
(*Pelecanoides urinatrix*)						
South Georgian diving petrel	Kerguelen	Molt			+	(Bocher et al., 2000; unpublished data)
(*Pelecanoides georgicus*)						

When feeding in the AZ, king penguins foraged primarily within the Permanent Open Ocean Zone (POOZ, Tréguer & Jacques, 1992), with some of them reaching the northern limits of the SIZ (Bost et al., 2004; Pistorius et al., 2017). Other species favor foraging within the SIZ, as indicated by the importance of Antarctic krill in the diet of Antarctic fur seals (Cherel et al., 2009) and of various procellariform seabirds (Cherel, Bocher, de Broyer, & Hobson, 2002; Cherel, Bocher, Trouvé, & Weimerskirch, 2002; Cherel, Quillfeldt, Delord, & Weimerskirch, 2016; Delord et al., 2010). Finally, some far‐ranging Kerguelen elephant seals transit through the POOZ and SIZ to feed within the productive but distant Coastal and Continental Shelf Zone (Labrousse et al., 2015). Consequently, population dynamics of subantarctic predators are shaped by Antarctic climatic indices (Barbraud & Weimerskirch, 2003; Le Bohec et al., 2008; Nevoux & Barbraud, 2006), making them at risk from natural or human‐driven environmental changes negatively affecting the biomass and distribution of Antarctic marine resources.

## CONFLICT OF INTEREST

None declared.

## AUTHOR CONTRIBUTIONS

YC and CAB conceived and designed the study. Prolactin measurement was performed by CP, and PB supervised stable isotope analysis. YC analyzed the data and wrote the manuscript, which was revised and improved by PB and CAB.
